# Approaching Challenges Posed by SARS-CoV-2 Genetic Variants

**DOI:** 10.3390/pathogens11121407

**Published:** 2022-11-23

**Authors:** José de la Fuente

**Affiliations:** 1 SaBio, Instituto de Investigación en Recursos Cinegéticos IREC-CSIC-UCLM-JCCM, Ronda de Toledo s/n, 13005 Ciudad Real, Spain; 2 Department of Veterinary Pathobiology, Center for Veterinary Health Sciences, Oklahoma State University, Stillwater, OK 74078, USA

In this new collection of the most viewed and cited papers, one of the Editor’s chosen articles, published in *Pathogens* in 2021, addressed the impact and the concerns relating to severe acute respiratory syndrome coronavirus 2 (SARS-CoV-2) and its variants [1]. SARS-CoV-2, which caused the pandemic of coronavirus disease 2019 (COVID-19), is an RNA virus discovered in December 2019 in Wuhan, China. Since then, the virus has spread worldwide with 6.61 million deaths reported by November 13, 2022 (https://www.worldometers.info/coronavirus/, accessed on 13 November 2022), a figure that is probably underestimated.

Vaccines have been the most effective intervention for the control of COVID-19, which, together with improved virus infection diagnostics, epidemiological surveillance, and treatment, have reduced disease prevalence and symptomatology (Figure 1). However, as reviewed by Janik et al. [1], the appearance of new genetic variants of concern and interest constitutes a challenge for disease prevention and control (Figure 1). Variants with higher transmissibility, and reduced effectiveness of neutralizing antibodies and other immune protective mechanisms in response to infection and/or vaccination, have affected virus detection and surveillance with increased severity of disease symptomatology and reduced effectiveness in disease treatment and vaccine efficacy [1].

Using the terms “SARS-CoV-2” plus “variants” to perform a search up to the date of November 11, 2022, PubMed recorded 1847 articles published in 2021 and 4529 articles published in 2022. These numbers highlight and support the relevance of the study of emerging SARS-CoV-2 genetic variants. The topics focused on emerging SARS-CoV-2 genetic variants and addressed in these publications include new treatment interventions (e.g., [2,3]) and vaccine formulations (e.g., [4,5]); virus detection tools (e.g., [6,7]); study of host–virus molecular interactions (e.g., [8,9,10]); virus infection, transmission, and lethality in vaccinated and non-vaccinated individuals (e.g., [11,12]); mechanisms of virus evolution and the emergence of new genetic variants (e.g., [13,14,15,16,17,18]); and vaccination efficacy and new strategies (e.g., [19,20,21,22,23,24,25]). 

Despite important advances in the characterization, surveillance, and prevention of emerging SARS-CoV-2 variants, challenges in other areas need to be considered for effective prevention and control of these genetic variants. Based on existing information, these challenges include the possible role of domestic and wild animal species in the appearance and transmission of new virus genetic variants, with impacts on both human and animal health (e.g., [26,27,28,29]), differences in the impact of environmental factors on virus persistence and transmission (e.g., [30,31]), and the still questioned role of arthropod vectors in virus transmission (e.g., [32,33,34]). 

Understanding genetic variability in SARS-CoV-2 and the functional implications of these mutations is important to understand the impact of new variants on virus infection and transmission, evasion of protective immune response, and disease severity. This information is also relevant for the design and implementation of personalized medical interventions and more effective vaccines. To approach these challenges, traditional well developed and novel challenging tools could be used.

Among novel tools, the quantum mechanisms may be considered to understand SARS-CoV-2 evolution and the appearance of new genetic variants and the design of vaccine protective antigens [35]. For example, quantum genetics could be applied to study virus evolution as proposed by Baianu [36] regarded as a “multi-scale process which is initiated by underlying quantum (coupled) multi-molecular transformations of the genomic and interactomic networks, followed by specific phenotypic transformations at the level of organism and the variable biogroupoids associated with the evolution of species which are essential to the survival of the species”. Regarding SARS-CoV-2, various quantum approaches have been proposed to study virus–host interactions [37,38,39]. For vaccine development, quantum vaccinology and vaccinomics approaches could be applied for the identification and combination of protective epitopes alone or in combination with post-translational modifications, such as glycans in chimeric antigens, to enhance vaccine efficacy against multiple virus genetic variants [40,41,42,43,44,45,46].

In conclusion, although the COVID-19 pandemic has been controlled, the appearance of new SARS-CoV-2 genetic variants is still possible, and a One Health multidisciplinary approach is required to be better prepared for the prevention and control of these viruses. Surveillance and timely communication of genetic analyses using validated and innovative quantum approaches is important to get ahead of the new virus variants. Vaccine design and implementation also needs innovation using quantum and other approaches to better protect against new emerging virus genetic variants. 

## Figures and Tables

**Figure 1:**
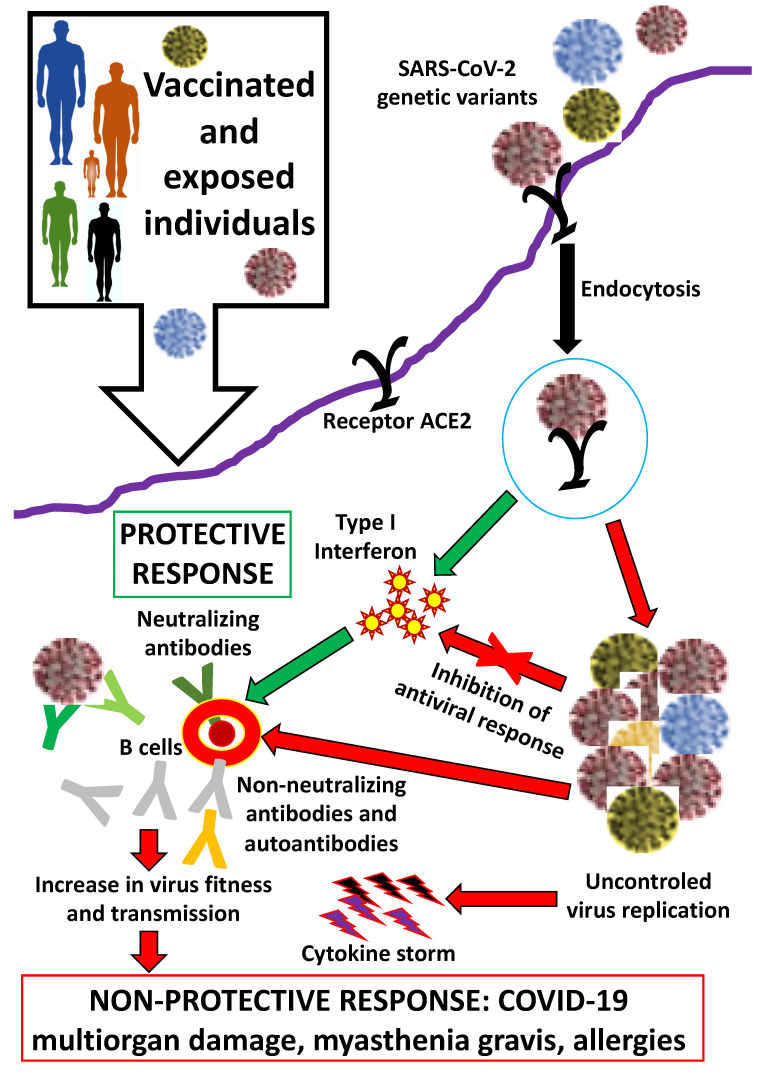
Impact of SARS-CoV-2 genetic variants on individual´s protective responses to coronavirus infection and vaccination and non-protective responses associated with COVID-19 prevalence and severity.
